# Being Able to Engage in Sports on One’s Own Terms: Positive Development in Sport for Older Adults

**DOI:** 10.3390/ijerph23050548

**Published:** 2026-04-23

**Authors:** Bartira Pereira Palma, Carine Collet, Evandro Morais Peixoto, Riller Silva Reverdito, Larissa Rafaela Galatti

**Affiliations:** 1 Faculdade de Educação Física, Universidade Estadual de Campinas, Campinas 13083-851, SP, Brazil; lgalatti@unicamp.br; 2 Faculdade de Ciências Aplicadas, Universidade Estadual de Campinas, Limeira 13484-350, SP, Brazil; 3 Escola de Educação Física, Universidade Federal do Rio Grande do Sul, Porto Alegre 90690-200, RS, Brazil; ca_collet@hotmail.com; 4 Programa de Pós-Graduação em Psicologia, Universidade São Francisco, Campinas 13045-510, SP, Brazil; 5 Faculdade de Ciências da Saúde, Universidade do Estado de Mato Grosso, Cáceres 78216-060, MT, Brazil; rsreverdito@unemat.br

**Keywords:** autonomy, aging, masters sport, mental health

## Abstract

**Highlights:**

**Public health relevance—How does this work relate to a public health issue?**

- The population aged 60 years and older is increasing worldwide, yet relatively few studies have examined sport participation among this group. This study provides scientific evidence that may inform the development of high-quality sport programs that are likely to promote health maintenance and well-being among older adults.

**Public health significance—Why is this work of significance to public health?**

- Aging is often framed as a negative process, and relatively few studies have examined practices that may contribute to the promotion or maintenance of health among older adults from a positive development perspective. This study highlights how sport programs can be structured to support health and well-being in later life.
- From a traditional perspective on aging, autonomy is often approached in functional terms, where engagement in sport or physical exercise is primarily understood as a means of maintaining functional capacity. This study offers a complementary perspective by showing how sport participation can satisfy the basic psychological need for autonomy, thereby supporting well-being and sustained engagement over time.

**Public health implications—What are the key implications or messages for practitioners, policy makers and/or researchers in public health?**

- Coaches and sport managers may consider the findings of this study when designing sport programs that support the satisfaction of older adults’ basic psychological needs, thereby fostering long-term engagement and its associated health benefits.
- Sport programs should emphasize not only the development of sport competence but also the promotion of healthy relationships and autonomy, enabling older adults to engage meaningfully with sport. Such approaches may contribute to sustained participation and to the health benefits associated with high-quality sport practice.

**Abstract:**

The aim of this study was to investigate older adults’ engagement in sport through the lens of the Positive Development in Sport (PDS), a framework aimed at fostering human growth in sport environments. This qualitative study involved 80 older athletes (M = 71.91 years, SD = 7.91; 45 women) engaged in regular sport practice and four experienced coaches (37–57-years-old). Data was collected across multiple contexts: brief in-person individual or small-group interviews during a competitive event; five in-person focus groups; and individual interviews. Data was analyzed using reflexive thematic analysis. Findings revealed a central theme, autonomy to engage in sport, supported by three subthemes: competence and confidence, health, and setting priorities. Participants described sport as a meaningful component of their identity, with sustained engagement driven by intrinsic motivation and harmonious passion. They reported increased self-awareness, intentional health management, and the ability to balance sport participation with other life domains, highlighting positive implications for mental health. Coaches who actively supported athletes’ psychological needs played a key role in fostering autonomy and personal development. Participants also emphasized the importance of inclusive relationships and pedagogical strategies tailored to older athletes’ goals and lived experiences. The findings suggest that sport in older adulthood can be a context for personal growth and mental health development.

## 1. Introduction

The practice of sport by older adults, particularly in competitive contexts, remains largely non-normative in contemporary society [1,2], which is related to the common association of aging with decline and limitation [3]. Sport is a cultural expression with significant potential to foster human development [4] and, therefore, a young people’s domain since development is frequently perceived as ending in adulthood [5]. The marginalization of older adults in sport settings raises important questions about age-related norms and underscores the relevance of investigating how older adults experience sport as a space for growth, autonomy, continued development, and maintaining mental health.

Mental health is a dynamic state of psychological functioning in which individuals are able to regulate emotions, cope with life demands and stressors, maintain satisfactory social relationships, and attribute meaning to their experiences, thereby sustaining adaptive functioning over time [6,7]. From this perspective, mental health is not limited to the absence of mental disorders, it also involves the presence of psychological resources that foster engagement, well-being, and social participation. Scientific evidence suggests that sport participation may contribute to mental health when it is organized within environments that support autonomy, promote experiences of competence, and foster meaningful social bonds, thereby strengthening personal resources [8,9,10].

Within this context, Positive Development in Sport (PDS) is a theoretical perspective aimed at fostering human growth and personal development in sport environments. PDS is rooted in the Positive Youth Development in Sports framework, an approach initially designed to support sport programs for youth. It challenges the predominant view that merely avoiding negative outcomes is sufficient for adolescent development, emphasizing the need to cultivate human strengths to increase the likelihood of success across various life domains [11]. Based on the understanding that positive development is both important and possible at all stages of life, this perspective has expanded to include older age groups [12,13,14]. Accordingly, we adopt the term PDS in this study to reflect a broader view that this approach is not limited to youth. Although research on PDS is growing, its application to older adults remains a particularly underexplored area.

Personal development in the sporting environment is not usually a short-term result [15] and, therefore, long-term engagement is a positive outcome of sport education, associated with benefits for physical and mental health [16,17]. Nevertheless, sustained engagement is most desirable when rooted in autonomy, enabling individuals to make choices that support continued involvement in sport [13,15]. People are more likely to engage, and remain engaged, in activities they endorse and identify with, feel competent performing, and that foster meaningful social connections. Under these conditions, individuals are better equipped to choose activities that allow them to regulate their participation in accordance with other life demands [18].

The Self-Determination Theory (SDT) offers a solid framework for understanding sport engagement. Its mini-theory of Basic Psychological Needs (BPN) [19] posits that individuals are inclined to seek activities in which their behavior feels self-directed and congruent with their wishes, and that resonate with their identity (autonomy), in which they feel effective and skillful while interacting with the environment (competence), and in which they experience connection, and a sense of belonging (relatedness). When these needs are met, they promote self-determined motivation and meaningful experiences. The satisfaction of BPN is associated with intrinsic motivation [20] and mental health indicators [21], representing an important focus of sport interventions.

Increases in intrinsic motivation over time foster the development of passion, understood as a strong inclination toward an activity that is valued, enjoyed, time-invested, and integrated into one’s identity [20]. The Dualistic Model of Passion distinguishes between two types of internalization: harmonious and obsessive. Harmonious passion arises when an activity is internalized autonomously, freely and integratively, without generating conflict with other life domains. In contrast, obsessive passion results from controlled internalization driven by internal or external pressures (e.g., social norms) and may lead to conflict with other areas of life. Contexts that support the satisfaction of BPN are more likely to foster harmonious passion and athletic identity development [22].

Recent studies have linked harmonious passion to positive outcomes in sport engagement. Verner-Filion and Vallerand [23] found that elite young athletes who reported harmonious passion and experienced satisfaction of their BPN also reported higher levels of well-being, better sport preparation, and, consequently, improved performance. In recreational sport contexts, Peixoto et al. [24] found that adult regular sport participants with intrinsic motivation experienced greater mindfulness than those with extrinsic motivation, and that both intrinsic motivation and harmonious passion were associated with higher mindfulness levels. Experiencing sport through intrinsic motivation and harmonious passion is linked to outcomes such as psychological well-being, mental health, and continued involvement [25]. Thus, understanding the psychological mechanisms underlying older adults’ sport engagement can inform practices aligned with PDS.

Despite growing interest, studies examining sport participation among older adults from a PDS perspective remain scarce [2,26]. Given that sport is not inherently developmentally beneficial, it is essential to investigate how sport experiences can be intentionally designed to promote positive development in later life. Research suggests that environmental factors can influence whether harmonious or obsessive passion is developed. Coaching styles that support the satisfaction of the need for autonomy tend to foster harmonious passion [27], since it fosters a healthy involvement with the activity; that is, individuals feel free to choose how to engage with the activity in a balanced way across life domains.

From an SDT-informed perspective high-quality sport programs are required for positive outcomes. Literature highlights the importance of structuring sport programs to include content related to (a) sport skills (i.e., tactical and technical), (b) personal and social development within the sport context (i.e., socioeducational), and (c) understanding sport as a cultural phenomenon and its broader social implications (i.e., historical-cultural) [28]. Such a comprehensive and meaningful experience is more likely to promote personal development and autonomous participation.

This study, therefore, seeks to address a gap in the literature by investigating Positive Development in Sport among older adults. It aims to contribute to our understanding of how sport environments can support continued involvement in later life, facilitating positive outcomes such as wellbeing and mental health. Given that physical activity and sport among older adults are often framed from a utilitarian perspective, as a means to prevent age-related decline, this study seeks to broaden the conversation by highlighting sport as a potential source of personal growth and satisfaction in later life.

The objective of this study is to investigate the psychological mechanisms underlying long-term sport engagement within the framework of Positive Development in Sport for older adults.

## 2. Materials and Methods

### 2.1. Study Design

Grounded in a social constructivist paradigm, this is a qualitative phenomenological study that explored the experiences of older athletes and their coaches in sports [29].

### 2.2. Participants

Eighty older adults participated in this study, recruited by convenience from three cities in the southeastern region of Brazil. Initial contact was made with coaches, who facilitated communication with team members. Participants were affiliated with private clubs and municipal sports centers. Participants’ sociodemographic characteristics, especially those of sports practitioners, and the number of participants per strategy are presented in Table 1. The group of Black individuals is subdivided into dark-skinned Black individuals and light-skinned Black individuals because, in Brazil, these different groups experience distinct consequences associated with racism. Therefore, institutions responsible for nationally representative population surveys in Brazil have adopted this strategy, influenced by the national Black movement [30,31].

Three male coaches and one female coach also participated, and their characteristics are described in Table 2. The volleyball and swimming coaches worked directly with the athletes interviewed in the focus groups. The badminton professional, however, was not the coach of the athletes who participated in the focus groups, since his team declined to take part. As a result, we interviewed a different badminton team.

### 2.3. Settings

#### Data Collection Strategies

All data were collected between April and September 2024. To achieve a comprehensive understanding of participants’ experiences and capture insights from multiple perspectives, three distinct data collection strategies were employed:

Short individual or small-group interviews at a competition event: Data were collected during one stage of a statewide multi-sport championship held across various regions of the state (JOMI—Municipal Older People Games). Interviews were conducted with athletes from adapted volleyball, swimming, athletics, table tennis, tennis, and choreography. Data collection included short individual (10–20 min) and small group interviews (15–25 min), as well as two in-depth interviews (37 and 51 min), conducted at the competition venue and athletes’ accommodation. Different interview strategies were employed in order to accommodate participants’ needs and competition schedules, in addition to facilitating the researchers’ understanding of the influence of the competitive environment on the perceived benefits of sport participation.

The researchers contacted the event organizers and obtained authorization to conduct data collection. With the organization’s approval, the first author and a doctoral student stayed at the same public-school lodging as athletes from two cities, allowing them to observe daily routines and build rapport by joining meals, leisure activities, and preparations for matches. Individual interviews were conducted by one of the researchers; in group interviews one researcher oversaw recording of audio and video. The researchers discussed the process during intervals to improve data collection. It is important to note that the researchers had no prior relationship with the group of athletes or coaches interviewed. Black athletes were intentionally recruited during this stage to ensure greater representation, as this was not feasible in the focus group sessions.

Focus groups: volunteers practicing individual and team sports exclusively in training groups were recruited from two cities in São Paulo state. Teams were recruited using a convenience sampling strategy and initially contacted through their coaches. All team members were invited to participate in the focus groups. Participants were eligible if they were aged 60 years or older, had been engaged in sport for at least one year, and provided informed consent to participate. Exclusion criteria included withdrawal from the study at any stage or withdrawal of consent for the use of recorded voice and video data. Locations were purposively selected to ensure socioeconomic diversity and varied motivations for participation. Focus groups were scheduled according to participants’ availability and conducted at training sites or adjacent rooms after practice sessions, except for Group 5, held in a café chosen by participants before opening hours and training. Five focus groups were conducted, organized as follows:

(1) Carried with members of a mixed-gender recreational adapted volleyball group training in a public facility in a socioeconomically disadvantaged area (i.e., typically operationalized through composite indicators of neighborhood-level structural inequalities, such as income, education, employment, and housing) [32]. Training occurs three times per week with minimal supervision; a Physical Education instructor provides warm-up and guidance on two of those days. Women have one dedicated weekly session, but most activities are self-organized.

(2) Carried with competitive swimmers (men and women) from a private sports club catering to affluent individuals. Coached by a woman, they train three to four times per week and participate in regional, national, and international competitions. Sessions are held alongside adult swimmers, with specific programs for older adults.

(3) Carried with members of a women’s adapted volleyball team training in a publicly funded facility in a socioeconomically advantaged area, twice per week (most train two more days per week in a different team), under a competitive framework and represent the city in regional events.

(4) Carried with members of a men’s adapted volleyball team training in a publicly funded facility in a socioeconomically advantaged area. They have a formal coach, train three times per week, and regularly compete in regional tournaments representing their city.

(5) Carried with a mixed-gender group practicing recreational badminton at a private non-profit sports facility for various age groups. They train twice a week with a female coach and compete locally. All participants also practice other sports, such as running and adapted volleyball.

Semi-structured individual interviews with coaches: conducted virtually, lasting between 60 and 90 min, at times chosen by the participants.

### 2.4. Procedures

Interview guides for both athletes and coaches were developed with open-ended questions grounded in the framework of Positive Development in Sports, specifically for masters’ athletes [1,5,12]. The interview guide was structured primarily based on the study by Dionigi et al. [12], for being one of the few that investigated the specific context of PDS and involving adults. The authors observed that sport facilitates the development of six assets in masters sports practitioners: competence and confidence, connection, commitment, cognition, and challenge. However, we included questions in the guide that allowed participants to express the particularities of their specific sports practice context. This was to ensure space for the particularities of the participants’ sports practice.

For athletes, the focus was on eliciting their motivations for engaging in sports, the perceived effects of this experience on personal growth and life in general, and the characteristics of sports practice that they valued. For coaches, the aim was to understand their perceptions of older athletes, the nature of their relationships with them, and the strategies they used to shape training and competitive environments, seeking expressions of behaviors that represent intentional promotion of PDS. The researcher took notes after interviews to help make sense of them during analysis. A second researcher helped with notes in focus groups. Focus groups and coach interviews were audio- and video-recorded, while short individual interviews were audio-recorded only. For short interviews conducted in pairs or trios, video was also captured. All interviews were transcribed verbatim for analysis. This research was approved by the local Ethics Committee; all participants signed informed consent forms and completed a sociodemographic questionnaire developed for this study. The names of participants and places mentioned are fictitious in order to protect participants’ privacy.

### 2.5. Data Analysis

Transcripts were analyzed using NVivo software (version 11) following a reflexive thematic analysis approach, considering its six steps: (1) familiarization, (2) coding, (3) generating initial themes, (4) reviewing and developing themes, (5) refining and defining themes, and (6) producing the report [33,34]. Analysis was conducted following a hybrid approach with deductive analysis based on a positive development theoretical framework, but also allowing for inductive elements, as we were open to singularities of this specific group of people. The coding process involved exploring semantic and latent meanings of transcripts as we tried to make sense of participants’ expressions, intentionally ironic statements, and other environmental observations in addition to what they reported.

## 3. Results and Discussion

We employed different interview strategies to understand the phenomenon from multiple perspectives. The data generated through these approaches were analyzed in an integrated manner; therefore, the results are presented jointly in this section, with indications in parentheses specifying the strategy from which each excerpt was derived.

The results of this study indicate that autonomy in sport engagement plays a fundamental role in participants’ long-term involvement. This autonomy is expressed in their daily decision to remain engaged in an activity that is meaningful to them and that they genuinely want to pursue. Through reflexive thematic analysis, we developed one overarching theme named autonomy to engage in sports, comprising three subthemes: competence and confidence, maintaining health, and setting priorities.

Overall, the findings demonstrate that sport occupies a central place in these individuals’ lives, and they highly value the athletic identity they have built. Mastering sport-specific competencies is part of a high-quality experience, as it allows them to continuously learn about the sport and about themselves, fostering deeper involvement in the activity. Their intense engagement in a sport they enjoy creates a favorable environment for maintaining health in a broad sense, mental and physical. These are experienced individuals who are in a stage of life that, at least for this group, affords them both financial and time flexibility, enabling them to prioritize the sport they deeply value. They do so wisely and harmoniously, without neglecting other important aspects of their lives. Together, these factors constitute the autonomy with which these athletes remain engaged in sport, not only because they want to, but also because they can.

**Figure 1:**
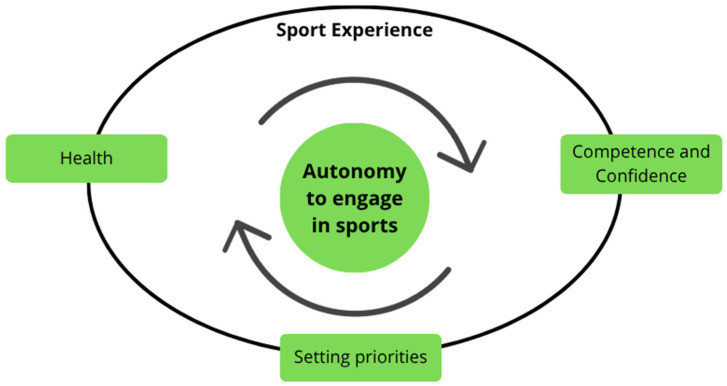
Representation of the findings of this study. Arrows represent the interaction among subthemes.

We draw upon the lenses of Positive Development in Sport, Basic Psychological Needs theory in combination with the Dualistic Model of Passion to discuss how the sport environment (i.e., structure, relationships, individual engagement) nurtures the autonomy demonstrated by these athletes, while their autonomy, in turn, supports both their own development and that of their peers. In Figure 1 we represent our findings, demonstrating that competence and confidence, health, and setting priorities, the subthemes discussed in this study, are stimulated by and stimulate the autonomy to engage in sports, illustrated by the arrows representing a positive feedback loop in which autonomy and sport-related factors reinforce one another. Inside the ellipse is the representation of the sport environment, and the rectangles positioned along the edge of the ellipse illustrate the influence of environments different from sport on these variables. For example, setting priorities that support continued sport participation may involve family support.

### 3.1. Autonomy to Engage in Sports

“That’s a good question. Because it makes me feel good” is what Alan (JOMI) answers to: Why do you do sports? Lúcia’s (focus group 5) comment also represents how most participants feel about being engaged in sports: “Sport is life for us, it’s life for me”. Gilson (JOMI) describes his reasons for practicing sports: “On the court I play sports, I exercise my body, right? And I fulfill myself as a person”. These individuals have wittingly chosen to make sports part of their lives due to the pleasure and sense of fulfillment they experience while practicing. They experience a sense of autonomy in their sports participation, perceiving themselves as having control over their engagement. Their involvement is facilitated by their perceived competence and confidence, which empower them to make the choice to participate actively in sports. Being physically and mentally healthy is important to decide and effectively accomplish sport participation; however, autonomy is mainly related to knowing they can engage in sports by their own means. Therefore, engaging in sports is a conscious choice achieved because they are healthy, have developed competence and confidence, and feel free to prioritize an activity they are passionate about.

Autonomy over one’s own involvement in sport and long-term engagement are positive outcomes predicted by models of sport participation, such as the Developmental Model of Sport Participation (DMSP) [35]. The DMSP outlines three possible pathways: one involving early sport specialization, which increases the likelihood of negative outcomes such as dropout; a second based on early sport diversification followed by specialization in adolescence, which may lead to elite sport performance; and a third, also based on diversification before specialization, but focused on lifelong participation. The latter two pathways are grounded in the development of personal and social resources that enable a healthy and long-lasting relationship with sport, increasing the likelihood of positive outcomes such as adequate physical and mental health and psychosocial development [16].

It was not the aim of this study to investigate which pathway within the DMSP participants had followed. However, considering that most of them have been involved in sports for extended periods (44 people for more than 20 years), even with interruptions throughout their lives, and that they report being satisfied with their current practice, experiencing benefits across multiple areas of their lives, we understand that we are observing a positive outcome of sport participation, long-term engagement associated with personal development.

Although we did not ask specific questions about the participants’ sport trajectories, some signs of sport diversification during childhood emerged. Thus, this study suggests that appropriate engagement in sport can indeed keep individuals active throughout their lives, even into old age. For example, Pedro, who is 73 years old and has been involved in sport for over 60 years, comments:

“I learned to swim when I was about 6 or 7. I swam competitively until I was 15 or 16. Swimming is my main sport. Then, for different reasons, I moved on to other sports, I was always into sports. I did karate for many years, played volleyball, ran, did all sorts of things.”

(Pedro, focus group 2)

These individuals’ sport experiences are deeply anchored in their sense of autonomy, both in choosing to remain engaged, which often involves making other important decisions in different areas of their lives, and in deciding how they want to engage, including the freedom to participate in different sports. As Marcia (focus group 5) shares: “For those of us who are new to badminton, when we manage to pull off a rally, we’re like, oh wow, we did it!”, expressing her sense of satisfaction in mastering a task within a new sport she was learning. The participants in this study thus demonstrate that their need for autonomy is being satisfied within the sport context, as their behavior demonstrates engagement driven by intrinsic motivation and passion, they feel confident navigating this environment independently and use the resources available to fulfill their aspirations and other BPN [19,36].

The development of intrinsic motivation for sport participation is closely linked to the development of sport competence, as the process of engaging in preferred activities and ultimately adhering to them is associated with the extent to which these activities support the satisfaction of basic psychological needs [19]. Activities that are perceived as sources of enjoyment and with which individuals identify may become objects of passion, thereby increasing the likelihood of long-term engagement and other positive outcomes, such as well-being [22]. Moreover, environments that support autonomy foster the development of harmonious passion [27], highlighting the importance of ensuring that older adults engaged in sport are able to exercise their autonomy and, for those who are just beginning their sport journey or starting a new discipline, that they have opportunities to develop autonomy in order to engage with sport on their own terms.

For participants to fully reap the benefits of sport engagement, the quality of the sport experience must be ensured [37]. The literature on sport pedagogy suggests that teaching-learning-training processes grounded in the principles of sport pedagogy—namely, the historical-cultural (linked to the sociocultural context of the activity), tactical-technical (related to sport competence), and socio-educational (associated with psychosocial development) domains—are well positioned to address the complexity of sport as a phenomenon and to foster greater autonomy among participants [28]. For example, when incorporating historical-cultural content into planning, coaches can recognize and critically reflect with athletes on the motivations behind older adults’ sport participation. While health maintenance is often a central motive, it should not be reduced to a strategy for avoiding physical decline, rather, it reflects a desire to remain actively and meaningfully engaged in sport, countering ageist assumptions. In the tactical-technical domain, coaches can prioritize the development of sport-specific competence, acknowledging that skill acquisition is not only a fundamental need for older adults but also a key factor in sustaining autonomous engagement in sport. Finally, by emphasizing socio-educational content, coaches can actively support the development of healthy interpersonal relationships and create a psychologically safe environment, one that encourages the growth of competence, confidence, and self-awareness. In doing so, they help create sport experiences that are developmentally enriching, socially supportive, and personally meaningful.

The coaches interviewed provided examples of strategies aligned with this approach. For instance, Laura described a strategy she used to counter the ageist stereotype commonly associated with her group of athletes, in an effort to value these individuals and aligning with a historical-cultural perspective:

“I called the River Club masters team the ‘experience masters’. I wanted to change it because I think ‘masters’ is tied to aging, not that I don’t accept aging, I actually think it’s wonderful. But I tried to give aging a new meaning. So, the ‘experience group’ is made up of people who’ve come this far, practicing sport with energy, with drive, so they’ve gained experience throughout this journey.”

(Laura, swimming coach)

Rafael explained how he plans competitive opportunities for the male volleyball team, whose members are still inexperienced and not yet accustomed to participating in competitive events. He recognizes the importance of these experiences in developing athletes’ competence and confidence, particularly with regard to tactical and technical skills:

“So, for the men’s team, since there’s no set competition except for JOMI, I try to organize round-robin tournaments, like the one we’re planning for August, so they get more used to game situations. You see someone in the stands watching, you see other teams there, there’s a referee, and you’ve got to figure out [how things work]”

(Rafael, volleyball coach)

### 3.2. Competence and Confidence

For the volunteers in this study, feeling competent proved to be crucial for meaningful engagement in sports, as it fostered a deeper and more enriching experience. Moreover, it enhanced self-confidence, which, in turn, further improved competence by encouraging greater openness to new and more complex sporting experiences. This cycle went through the increase in self-knowledge that exposure to more complex sporting experiences provided, which helped them to gain more confidence and competence, ultimately increasing autonomy for engaging deeper in their sport and in new sports as well.

Competence and confidence are reported together because participants’ reported experiences show an association between the constructs. Marcias’s comment illustrates this association:

“And this thing about personal enjoyment, you know? In swimming, there was something I had never done until recently, I mean, I dive, but I had no idea how far it was from one lane to the other. And there’s this drill where you have to swim under the lane. That was a challenge for me, because you’re not looking up, so it’s hard to know when you’ve crossed the lanes. But I learned, and it was something personal for me. Now, I do it easily.”

(Marcia, focus group 5)

Dionigi et al. [12] investigated competitive sport participation among adults and older adults and identified six personal assets that contribute to participants’ personal development. Similar to the findings of the present study, competence and confidence were among those assets. Likewise, Kirby and Kluge [14] found that older women volleyball players experienced empowerment through improved sport competence, which closely resembles the experience of self-confidence. This study adds further evidence supporting the relevance of competence and confidence in sport for older adults, challenging ageist beliefs that suggest sport programs should not focus on these variables for this population. Participants’ narratives reinforce this interpretation, as illustrated by João, who describes how he feels after completing a swimming session.

“Swimming, when practice is over, makes you feel uplifted. It is different from other sports, I don’t know why. We feel empowered when we complete the training session here. Even if you are not training with the coach, you may still swim a little on your own, something like that. You completed your task of swimming 1500 m, and you come out of the water feeling enormous.”

(João, focus group 2)

For João, the experience that swimming provides is unlike any other sport. However, it does not seem that the sport type itself is inherently different, but rather that the feeling upon finishing the session stems from a sense of accomplishment, the perception of having been capable and competent in swimming the distance prescribed by the coach. Francisca (focus group 3) participates in the municipal master’s volleyball team and also expresses a sense of competence when she states: “And you are not selected [for the volleyball team], right? If you are not good, you don’t make it.” Francisca’s teammate Carla (focus group 3) expands: “And there is another thing, you have to strive because only the best goes to the competition. Do you understand?”

In contrast to the study by Dionigi et al. [12], in which the sport environment supported the maintenance of competence and confidence but was more closely associated with the preservation of physical function rather than sport performance, many participants in the present study emphasized that playing or swimming well is highly important and gives meaning to their engagement in sport. In this study, participants strongly associated competence with sport performance, expressing a sense that they are capable of doing it, and a confidence in their mastery of the skills needed to effectively interact with the sport environment. This behavior indicates that their basic psychological need for competence is being fulfilled.

Competence satisfaction can stimulate interest and enjoyment in the activity in the sport environment, which can lead to engagement [38,39]. To satisfy this need, individuals tend to engage in activities that demand slightly more than their current abilities, seeking challenge [36]. In this regard, Henrique shows confidence in his competence and emphasizes the importance of feeling challenged to improve his skills. The challenge motivates him to train and enhance his performance.

“I think I play wonderfully well, but many times my ‘well’ is relative. For example, in a competition, you face high-level opponents. Even though I believe I play well, the opponent is often superior. So, you have to train more.”

(Henrique, JOMI)

Vitor (JOMI) also expresses feeling challenged in his sport: “I feel [challenged] to train in order to improve, to observe the opponent, where they succeed, so I can try to achieve the same success or neutralize their play. So, there is a huge challenge in table tennis”. Meanwhile, Luís describes his feelings with the constant learning of new skills in his sports routine: “I feel fulfilled. We are always learning, every day is a learning experience. Sometimes, this learning happens involuntarily; you are playing, and suddenly, you discover a new move that you had never thought of before”. The challenge exposes these individuals to new situations that contribute to the development of their sports competence, which, over time, increases their confidence in their own athletic abilities. This is experienced in competitive events, which have an important role in these individuals’ sport and personal development, but also in daily practice and recreational situations. Marcos’ account serves as an example:

“I once went to another place, and the people there invited me to play. I had no hesitation; I stepped in and played with them. Later, they came to me and said, ‘Wow, that was great! Come play with us again.’ That, for me, is truly rewarding, gratifying. It’s completely different from just standing there, watching everything without participating. So, the little I know, the little I can do, gives me the confidence to join in and have fun with them.”

(Marcos, focus group 1)

Marcos explains how his sports competence enabled him to exercise his autonomy by engaging in sports of his own volition and accepting his peers’ invitation to play in a recreational setting. His attitude demonstrates trust in his own ability to engage in the game, and this openness allows him to expose himself to new and more challenging situations. In this case, Marcos perceived himself as competent to spontaneously engage in a sport activity; his self-assessment was confirmed by the effectiveness of his performance and by the feedback he received from his peers, contributing to the satisfaction of his need for competence. These individuals’ sporting experience and maturity enable them to be aware of their actual competence and to trust in what they can or cannot do, as Henrique explains:

“Because, in reality, here we meet people, Thiago, for instance. Thiago was a Brazilian champion; we have a South American champion, a São Paulo state champion, and they all participate in JOMI. So, competing against them is very difficult, but they are not invincible.”

(Henrique, JOMI)

Thus, the development of competence is linked to mechanisms that promote the valuing of sport and the individual’s confidence in their ability to practice it, ultimately leading to the integration of sport into the individual’s identity. Moreover, feeling capable of performing the activity contributes to athletes perceiving their engagement as autonomous, that is, as a personal decision based on their own perceptions [19].

The athletes interviewed have already undergone the sport initiation process and demonstrate sufficient competence and confidence to remain engaged in their practice. However, their accounts suggest that they interact with teammates who possess varying levels of competence, which at times leads to conflicts, as reported by Gustavo (focus group 5): “So if I make a mistake, I like Lucia because she doesn’t say anything, but then there are others, other people yell at you, and that’s what’s bad.” Thus, the sport environment can be a fertile ground for feedback that may influence the satisfaction of the need for competence either positively or negatively [39]. Marcos (focus group 1), for instance, described a situation involving a teammate who was excluded by the group: “She was not included in the game; she was not passed the ball; and eventually gave up participating”. It is worth noting that Marcos is part of a volleyball team that does not receive specialized coaching. While having a coach would not guarantee improved relationships, as this would depend on the coach’s approach, these individuals perceive that it could contribute to an environment that better supports quality relationships, an idea supported by the literature [37]. José’s account illustrates how they believe a coach could help address a gender prejudice-related conflict within their mixed-gender team:

“There’ve been fights here, real fights, almost got physical, you know? So, we really need a coach here to be at every practice, because we’ve got some powerful people here—the women. Everyone used to play together, women too, there were like three or four teams. Then they split things up because of fights, lack of respect, stuff like that. So now the women play on Tuesdays and Thursdays. What we need is a coach here to either guide us on the basic rules or just give us some encouragement.”

(José, focus group 1)

In this regard, coaches play a key role in providing an environment that supports the satisfaction of the need for competence in sport [38,39] with an emphasis on intentional and well-planned actions, thereby increasing the likelihood of positive experiences [28]. The development of competence involves building high-quality relationships among athletes, in which they support one another [39], as well as fostering a safe sport environment where making mistakes is acceptable, and athletes feel comfortable taking risks and testing their abilities.

Coaches contribute to the development of competence and confidence by treating these athletes as the capable people they are and by giving their sporting practice the necessary seriousness. In this way, they contribute to the development of autonomy over one’s own practice and to the satisfaction of the need for autonomy [19,22]. Laura (swimming coach) teaches her team all the skills they need to be competitive swimmers: “All the nomenclature that is used in competitive swimming is also used by my master’s team. So, they know what they are doing”. Rafael (volleyball coach) is concerned about his athletes’ learning and explains how they demand an active attitude on the court: “So, they feel important, and they actually want to be pushed. On the days when I just pass by during practice and don’t say anything, they’re like, ‘You didn’t say anything today? I’d rather you push us”.

Perceived competence and confidence are linked to the sport they identify with the most. Although once they have confidence in their skills, they are open to participating in different sports, they often have a preferred sport, in which they invest time and energy to improve their skills and win competitions. This aligns with the concept of passion, as athletes invest more energy in the sport for which they have developed strong feelings [22], and represents important information for coaches, since they can tailor the sport intervention knowing the intensity of investment to expect from each athlete, and the demand for performance that would be more adequate in each case. For example, Felipe is a volleyball player and a runner, and when questioned about the importance of participating in competitive events, he answers:

“In volleyball I don’t want to, I just want to learn. I don’t aim to be a champion, nothing, I want to learn. Now, I do athletics and street running. Then, yes, I enter to compete, I enter to win, right?”

(Felipe, focus group 4)

Felipe identifies with running, but in volleyball, his goals are recreational. Paulo tells a similar story:

“As for running training, I talked to other coaches, they taught me how to run, how to move my hands, my legs, everything, but I was never really that concerned about winning a race. I like winning volleyball, I like winning soccer, but I never dreamed of winning a race. Sometimes we are happy to have achieved the best time in our age group.”

(Paulo, JOMI)

Therefore, it is important for coaches to be attentive to the particularities of their teams, in terms of goals and perspectives, as well as to the individual characteristics of each athlete, in order to create an environment conducive to the development of competence [38,40]. This involves recognizing that a defining feature of the sport environment for these individuals is the diversity of goals, regardless of context. In one team competing at a state-level championship, therefore participating in competitive sport, we observed athletes whose main focus was not on winning. As Amanda (JOMI) explains about her participation in the competitive volleyball team: “I’m not that kind of person who’s out for blood.” However, when asked how she feels playing tennis, Amanda says, “Oh, I love beating the other person”.

Closely linked to competence and confidence development, sports participation allows athletes to gain self-awareness while practicing sports, which, in turn, contributes to building confidence. This process fosters openness to new experiences and a deeper understanding of the role of sports in their lives. The establishment of tailored self-assessment parameters is highlighted as a crucial strategy that most athletes utilize to better comprehend their own competence. It does not make sense to these individuals to compare their performance with that of their opponents or teammates. However, winning is important as well; it is part of sport and the recognition of their effort.

Luís demonstrates self-awareness by describing the skills he has recently developed and what he believes has contributed to the improvement of his table tennis game.

“Yes, agility and a greater diversification of my move. And I think that what also contributed to this agility, not only the tennis practice, but also the fact that I was going to the gym, this helped in the development of my body so that I could be agile in the game.”

(Luís, JOMI)

These athletes demonstrate refined awareness of their own skills and limitations developed over the years of sports practice, as Cecília reports when commenting on what she learns from participating in competitions:

“I also see my limits, my achievements through competition, which can have a positive and negative side. At one time I tried running and, even because of my body type, it was a daily struggle to improve. […] I got frustrated because I wasn’t performing well in that sport. So, I think that competition, for me, is very good. But it can also bring frustrations when you don’t perform and choose the wrong sport for your body type, for your category. I think that this has to be taken into account.”

(Cecília, JOMI)

By recognizing their potentials and limitations, these individuals set personalized goals that help maintain a sense of challenge, enhance their competence and confidence, and ultimately lead them to feel good about themselves, which may contribute to their mental health, as explained by Janaína and Roberto.

“For me, I always compare my results. Like, last year, we went to a race in Campinas, and that same race happened again last Sunday. So, I check, what was my time last year? Oh, it was this. And this year? Oh, I improved! So, competition gets to us.”

(Janaína, focus group 5)

“The most important thing is not the position I got, but the goal I set. So, tomorrow, for example, I’m going to run the 1.500 m, and I’d like to run for 5:40, right? I’d like to run smoothly […] without showing any signs of suffering or tiredness. Because, […] I want the audience to look at me and say, “Wow, the guy does what he likes. That’s the best place for him at that moment.” So, if I can achieve the time, then I’ve already reached my goal.”

(Roberto, JOMI)

Gilson, a field tennis player, demonstrates not only that he recognizes his limits, but also that he uses strategies to continue practicing sports while acknowledging his current skills and possibilities.

“At the club I go to there are competitions that I avoid participating in, to avoid injury. I play more doubles to avoid injury, to extend my period of playing sports.”

(Gilson, JOMI)

These athletes are deeply engaged in their sport activities, in a process of personal involvement that enables intentional effort toward self-awareness [40]. This process is important because it allows individuals to understand their true capabilities and potential, thereby opening themselves up to new experiences and exposing themselves to challenging situations that contribute to the development and refinement of sport competence. This process is closely related to the satisfaction of the basic psychological need for autonomy, which these individuals express through autonomous behaviors of sport engagement, as it enables self-regulation [18], that is, the ability to recognize personal limits and determine how far those limits can be stretched, thus avoiding excessive frustration due to failure or lack of challenge.

The continuous process of developing competence and self-confidence allows these individuals to choose to engage more deeply with the activity they value [18], gradually gaining ownership of the sport environment as they learn to navigate it with maturity, making use of available resources to meet their own needs. Being aware of their own skills and potential helps them recognize that their sport-related choices stem from within themselves, fostering the development of intrinsic motivation and harmonious passion for the activity, which are associated with positive outcomes. Passion, specifically, sustains dedication to practice, with the investment of time and energy in self-perception and the development of sport competence [22].

The literature suggests that the development of sport competence is tied to the provision of adequate structure, which includes providing consistent rules and objectives, guidance and support, as well as appropriate feedback [41]. A quality structure also involves the use of appropriate pedagogical strategies, organizing tactical-technical content systematically and with increasing complexity, considering the characteristics of each athlete group [28,37]. Additionally, by supporting the development of autonomy, coaches also facilitate the satisfaction of needs for competence and relatedness, as individuals begin to make meaningful choices and invest in strategies that support the fulfillment of these other needs [18]. Thus, coaches can allow athletes to take risks, make choices, be heard, and actively participate in the activity, fostering an environment that supports the need for autonomy [22].

Moreover, building a strong coach-athlete relationship, grounded in trust and genuine care, can foster not only the development of supportive peer relationships among athletes but also the enhancement of sport competence and the fulfillment of the need for competence [38,39,40]. The findings of this study suggest that possessing a high level of sport competence is a crucial factor in exercising autonomy in sport engagement, as it is associated with sustained involvement and the ability to make self-directed decisions regarding one’s practice, such as learning a new sport or participating in spontaneous activities. Therefore, it is essential that coaches provide older athletes with genuine opportunities to develop their sport competence.

### 3.3. Health

Being engaged in sports is a way of staying healthy, of taking care of themselves by fostering the psychological, physical, and social aspects of their health. Being healthy is the only way to continue practicing sports and winning competitions; thus, practicing sports for a long time fosters the discipline to take good care of one’s health. However, health is not central to their sporting experience as it represents only one of the important aspects of being engaged in sports.

The association between engagement in competitive sports practice and physical and psychological health is recurrent in the literature [2,12,42]. The participants in this study reinforced these findings, as demonstrated by José (focus group 1): “My cardiologist has been treating me for over 40 years. I follow his advice. [He says] Keep exercising. Because when I go there, my cholesterol is good, and the rest is good too”. This demonstrates how important the maintenance of physical health is for these individuals and that the sport environment fosters discipline and motivation to take care of their health and to maintain their body at full capacity to keep practicing sports and living their active lives. Carlos explains how being engaged in sports helped him to take care of his health:

“It’s a source of motivation, which serves to maintain physical health, keep focused and it helps a lot with mental health. Those who practice sports mainly develop discipline, that is essential to maintain physical health and, especially, mental health. […] You can’t afford to neglect your health. So, you are required to keep your physical health up to date in order to compete.”

(Carlos—JOMI)

Carlos expands the concept of health including mental health, and he is followed by Maria (focus group 2), who links her practice to mental and social aspects of health: “It’s a very powerful and very important antidepressant that helps, it helped me personally, it helps me a lot. I think that this group is very friendly and everything, it has this power”. Fátima has a son with disabilities and is his full-time caregiver. For her, participating in sports is a way of maintaining her health, as she explains:

“Well, for me, it’s my life, you know? I value myself a lot, I take great care of myself so I don’t get hurt. Because I always think, if I get hurt, my God, the only thing I do in life is take care of my son and play tennis. I love it. For me, it’s about health, it’s mental, it’s physical, it’s about friends, social life, it’s everything.”

(Fátima, JOMI)

Moreover, taking care of one’s health is seen as a way to continue practicing sports and competing, as Carlos explains:

“There are several sports that really require you to be in good physical shape. So, to be able to take part, the person has to take responsibility for their own health. Sport promotes health, and it helps people realize how important it is to keep their health in check.”

(Carlos, JOMI)

This broad understanding of health is complemented by Luisa (JOMI), who makes a connection between health and happiness, demonstrating how being a sportsperson helps her navigate this stage of life: “So, I say, for whom practices sports, regardless of the modality, the sky is bluer, life is more poetry. Understand this! I am happy and joyful because I practice sports and because I have never been sick”.

The literature indicates that health is indeed a key motivator for sport engagement among adults and older adults [42,43]. There is no doubt that regular sport participation is associated with health, and the positive effects of an active lifestyle on the physical and psychological well-being of older adults are well established in the scientific community and widely recommended by health professionals, organizations [44,45], and the media. These benefits are corroborated by the athletes interviewed in this study, who report physical vitality, enjoyment, happiness, and meaningful social interactions, among others. In line with Dionigi et al. [12], participants in this study appear to perceive themselves as being in control of their own health and competent in implementing important strategies for maintaining physical health, such as staying active, attending regular medical check-ups, and maintaining a healthy diet.

An important point is that in Dionigi et al. [12], the authors included health within the theme of competence and confidence, as they associated sport participation primarily with physical health and the ability to perform motor tasks, often unrelated to sport, which gave individuals a sense of control over their bodies and reduced the likelihood of age-related illnesses. In the present study, however, although participants made similar comments, they did not directly associate health with competence in their narratives. It is common to assume that older adults engage in sport for fun and physical health maintenance, as the pursuit of performance is not typically expected of older individuals, reflecting an ageist understanding [1]. The findings of this study point in a different direction, reinforcing the importance of regular sport practice for health among older adults, while also emphasizing the relevance of sport-specific content. This confirms the status of sport as a complex cultural phenomenon that cannot be reduced to a single function, such as serving merely as a tool for health promotion [46].

Although participants expressed satisfaction with their health and attributed this to being athletes, they also demonstrated extrinsic motivation in this regard [18], often referring to validation from doctors and family members. As Vitória (focus group 3) recalls about her physician: “He said, ‘You inspired me.’ He didn’t forget. I went back and he said, ‘I know who you are—the volleyball player who inspired me, and now I’m running twice a week’.” The norm of regular exercise or sport practice as a marker of healthy aging is widely accepted, which can lead to the stigmatization of those who are not physically active [47]. For most participants in this study, the first response when asked about their motivation to engage in sport was “health,” as if to affirm they are doing their part to age well, distinguishing themselves from inactive individuals, as suggested by Francisca (focus group 3), who makes a comment that clearly distances her from the stereotype of frailty, even though her ability to perform a simple task such as standing up from a chair is unsurprising given her high level of physical functionality: “She (her dentist) is amazed how at our age we don’t need to hold on to anything to stand up. I just get up in one go”. Thus, although it is evident that health is a relevant factor in older adults’ sport engagement, participants also appear to strive to meet social expectations related to healthy aging.

According to the World Health Organization, the current concept of health encompasses physical, mental, and social well-being and explicitly acknowledges that health is not simply the absence of disease [48]. The participants in this study demonstrated a broad understanding of health, aligned with the WHO’s definition. This is significant given the common association between physical activity in older adulthood and the prevention of physiological decline [2]. Designing sport programs for older adults based solely on this logic reflects ageist assumptions and results in impoverished experiences, with limited potential to meet the actual motivations of participants or satisfy their basic psychological needs. Consequently, the likelihood of fostering intrinsic motivation, harmonious passion, and personal development is reduced [40,46]. Likewise, reducing sport to a mere tool for health maintenance can contribute to the moral judgment of individuals who are unable or unwilling to engage in sport. Such assumptions fail to consider the social dynamics that limit or prevent access to sport for older adults, including socioeconomic factors, making it unjust to blame those who are already marginalized due to lack of access [45,47]. By taking these factors into account, coaches and other professionals can help foster a healthier and fairer sport culture, one that is more conducive to positive outcomes.

The findings of this study, therefore, suggest the need to move beyond the assumption that older adults engage in sport solely for health reasons. Rather, health is one of several motives [1]. Recognizing this supports a more holistic understanding of the individual and affirms their capacity to develop intrinsic motivations for sport participation, which in turn fosters autonomy, the ability to make self-determined choices based on personal values [18]. Coaches can help athletes develop resources such as strategies for managing their health autonomously. For example, Rafael (volleyball coach) notes: “I give them the game schedule, and I create a training plan with what’s appropriate for them to work on at the gym”, since his team does not have physical conditioning sessions during regular practices. And each athlete is thus responsible for managing their own physical preparation. Laura (swimming coach) contributes to athletes’ satisfaction and personal fulfillment by incorporating their sport achievement goals into her planning. This may enhance mental health by improving self-esteem and fostering the development of autonomy in sport, as it supports both competence and confidence [9].

### 3.4. Setting Priorities

In this stage of life, athletes have more autonomy to choose to prioritize sports in their lives. If they had children, they have grown, and they are now mostly retired or have a flexible working schedule, leaving them with more time to engage in different activities they like. Maturity allows them to put sport in a healthy place in their lives. They know exactly what is worth and what is not, and their families and friends come first. Deeply understanding the value of their relationships, they prioritize them, which means that winning at all costs or insisting on a discussion that is not important is not worth it.

One important marker of the aging process is retirement, reduction of the professional workload, or the schedule flexibility that financial stability allows in this stage of life, at least for those with socioeconomically advantaged backgrounds (i.e., with high incomes), who can decide to retire or reduce the workload. The phenomenon is perceived as a sense of being lost, as Marta describes:

“[…] my surname was Hospital L. When they remove this surname, who am I? (referring to her retirement). Yes, that leaves a big void, and at that time, sport, swimming, specifically, this welcoming group, this special group, entered the story very well.”

(Marta, focus group 2)

Leonardo continues and explains his understanding of this phenomenon.

“So, one concern I had when I was retiring was, well, and now what to do, right? And we moved here near the club, and then there’s stuff to do, play sports, do volunteer work, right? It also gives you a sense of belonging, it’s like what Marta said, so I stopped working to earn money, but now I’m participating in an organization, in something that… Ah, being part of the swimming team, swimming, takes up a little time, and you have goals, you have new friends, so it helps you fill your days in a different way than you were used to.”

(Leonardo, focus group 2)

The choice to engage in sports due to the increase in free time related to retirement was true for men and women, but the one related to reducing child-rearing tasks was mostly reported by women, as demonstrated by Carla and Teresa:

“I’m 81 years old and have been playing sports since I was 61. So, I’ve been playing volleyball for 20 years. When I was young, I used to swim, but then I had kids, and I stopped for a while. Then, since I had a job that kept me busy, I stopped for a long time. When I was 61, I was invited to play volleyball. I liked it and I still do it today.”

(Carla, focus group 3)

“When I was 14 or 15, I represented my city in the games, which today are the Jogos Abertos [Open Games], I did high jump, long jump, and run. So, I’ve always been involved in sports. Then you get married, life changes, you have children, you have a lot of things, your sport is inside the house.”

(Teresa, focal group 3)

These findings reinforce statistics indicating lower levels of engagement in physical and sport activities among women during youth and adulthood, which are associated, among other factors, with social roles predominantly assumed by them [45]. It is interesting to note that none of the interviewed men mentioned the increased free time due to the fact that children had grown when explaining their involvement with sports.

The participants interviewed have chosen sports to fill their free time because it is an activity they deeply enjoy. Although it is not possible to establish a cause-and-effect relationship due to the cross-sectional and descriptive nature of this study, the findings support the propositions of the DMSP. Most participants reported engaging in sport during childhood, which may have contributed to their return to sport in adulthood, as early sport involvement is associated with the development of physical literacy, an important factor in perceiving sport participation as a viable option later in life [49]. Artur’s account illustrates a break in sport participation due to work and a confident return to multiple sport modalities in adulthood:

“I really got back into training more seriously around the year 2000, after getting scolded a bunch of times by doctors who told me I was neglecting my health and had to do something—I was overweight. So, I came back here and spent a good while just doing fitness stuff, because that’s what I could manage at the time […]. Then, when I retired, Laura invited us to join the masters team, and I’ve been on the masters team for over 15 years now. So, I’ve been going along with her, and I’m still playing basketball here at the club.”

(Artur, focus group 2)

In the current stage of their lives, the free time they have, coupled with the maturity they developed over the years, along with the financial stability for some of them, allows them to make life choices and organize their schedule prioritizing sports, but also considering that it is not the only thing in their lives. For example, when questioned about her experience when organizing her schedule to practice sports, Cecília says:

“No, because I prioritize sports. I have this possibility. So, I work part-time to be able to fulfill my sports obligations. I’m financially ok now, so I don’t need to do much [so] I started to do sports.”

(Cecília, JOMI)

Laís, interviewed shortly before her table tennis match, can easily organize her routine to participate in practices and competitive events, and she does it willingly:

“I prioritize sports. For example, I was coming here this week [to the championship], so I decided to stop doing housework and leave things behind because I don’t have kids. And let’s dedicate ourselves to sports. Let’s rest so we can get there well prepared physically and psychologically. It’s a sport where you have to think a lot before you put the ball on the table.”

(Laís, JOMI)

Tales, who was playing in the same championship, plays sports for fun. He has no intention of playing more seriously than he already is. This is exactly the space he wants his sport to occupy in his life.

“I don’t want to go out and compete. I’ve done a lot, I’ve participated in state competitions, I’ve participated in regional competitions. And we spend a lot of time [to do this], and family takes a back seat. So, I prefer to train table tennis on Wednesdays and Fridays and spend the weekend with my wife and daughter”.

(Tales, JOMI)

Likewise, Angela says:

“Volleyball is not the only thing in my life. I, for example, love to travel […] I can’t focus my life on just one thing. I think it’s even good that you have other interests and enjoy doing other things.”

(Angela, focus group 3)

Angela and Tales participate in competitive sports and experience this in harmony with other aspects of their lives while still letting sports occupy a prominent space in their routines, which could be interpreted as a harmonious passion for their activity, given their intense engagement with their sport while balancing other life activities [22]. Marcia explains how she now balances house chores to prioritize sports:

“I think that as we grow older, I think we know how to prioritize things better. You know, we start to prioritize things in a different way. Oh, if I leave the dishes now, nothing will happen, I can do this thing and the dishes will be there, I’ll wash them later.”

(Marcia, focus group 5)

Even though they still take care of their families, at this stage of life, this is more easily integrated into their lives and more supported by their relatives. Francisca cultivates her relationship with her family and takes care of herself wisely.

“I have a grandson. So, I’ve been taking care of him since he was little. But my daughter doesn’t let me miss training and games. I put all the games I have during the year on her fridge, so she respects those dates, you know? She tries not to schedule meetings on those dates.”

(Francisca, focus group 3)

In addition, these individuals cultivate their sporting relationships with wisdom. Their friends are an important part of the reason they practice sports. Thus, it makes no sense for them to put unimportant conflicts ahead of the relationships they have so carefully cultivated. Vitor describes that, contrary to common sense, sport is competitive for them, and conflicts exist:

“No, it’s a… someone makes a play and thinks that the ball, for example, hit the net and it didn’t, and so on. Sometimes there are conflicts. Obviously, in our age group, these conflicts are very limited. Every now and then, there’s a fight going.”

(Vitor, JOMI)

Then, when asked about how these conflicts are solved, he answers: “Nothing, most friends just hush it up. It ends like that; it ends in nothing.” (Vitor, JOMI).

They practice sports with responsibility, making intelligent choices towards a healthy sport engagement, choices that do not disregard other important dimensions of their lives. BPN theory posits that people have the tendency to engage in activities that they experience as enjoyable and that will favor the satisfaction of psychological needs [19]. For one or more of these activities, individuals will demonstrate passion, that is, a tendency to become intensely involved in an activity they highly value, perceive as important to their self-concept, and into which they invest significant time and energy [22]. The participants’ narratives demonstrate that they are passionate about sport, which has come to occupy a central place in their lives and become an essential part of how they define themselves and construct their identity [22].

The way sport has been integrated into these athletes’ identities, grounded in a strong and consistent appreciation developed over years of involvement, suggests an autonomous internalization of the activity, and therefore, a sport experience rooted in harmonious passion. In this condition, these athletes manage their sport participation in a balanced way, that is, without excessive conflict with other areas of their lives. Participants’ descriptions of how they harmoniously organize their various life tasks indicate that sport does not occupy an overwhelming place in their routines [22]. Therefore, it is important that the sport experience supports the satisfaction of the needs for competence, relatedness, and autonomy, as this contributes to the activity being valued by the individual and integrated into their identity through the development of harmonious passion, ultimately fostering a meaningful and enriching experience that promotes personal development.

This way of relating to the activity is not universal, as the group of older adults who practice sport is not homogeneous. There are reports of an excessive focus on winning, which can lead to conflicts and the exclusion of less skilled individuals. Therefore, coaches should be attentive and implement strategies that incorporate socio-educational content, such as emphasizing respectful and supportive interpersonal dynamics. They may also draw on historical-cultural content, which can help foster reflection on the social construction of sport as a hypercompetitive environment reserved for highly skilled individuals [28]. In this way, coaches can create an environment that supports the satisfaction of the needs for relatedness and autonomy. When individuals feel meaningfully connected to their peers, the likelihood of continued participation and a sense of belonging increases [50], and by fostering ways of engaging with sport that are more inclusive and prosocial, coaches increase the likelihood that more individuals will choose to participate and remain involved, thereby strengthening the sport system for older adults. This also enhances the chances of satisfaction with the activity and personal endorsement of their engagement, moving toward an autonomy-based involvement [18].

## 4. Practical Applications

Sport coaches and managers can use the findings of this study to design high-quality sport programs, which include properly developing tactical-technical content and creating environments that support the development of competence and confidence, highly valued by the participants. To achieve this, coaches may implement training strategies that prioritize sport-specific skill development and provide opportunities to compete at levels appropriate to each group’s skills, thereby enhancing learning and self-awareness.

A favorable environment for developing competence and confidence also requires supportive and encouraging relationships that foster a safe space for taking risks and making mistakes without judgment. Therefore, coaches should invest in strategies that promote healthy coach-athlete and peer relationships through socio-educational content. Furthermore, coaches must ensure that athletes have adequate access to information and resources to care for their health, encouraging regular medical checkups, proper physical preparation, the cultivation of friendships, and personal satisfaction in sports practice. In doing so, coaches should avoid framing sport solely as a tool for physical health maintenance, which diminishes the significance and complexity of sport in these individuals’ lives. The historical-cultural dimension can serve as a valuable framework for reflecting on these issues with athletes. As a foundation for their professional practice, coaches should respect the wisdom and maturity of older athletes, allowing space for them to exercise autonomy in their sport participation by acknowledging their suggestions and needs. By fostering autonomy-supportive environments that nurture competence, relatedness, and self-endorsed engagement, coaches create the interpersonal conditions necessary for the development of harmonious passion.

However, the cultivation of autonomous engagement in sports does not depend solely on individual coaching practices; it is also shaped by broader structural and cultural conditions that define who has access to sport and under what circumstances. Sport is generally not regarded as a context conducive to older adults’ development and fulfillment of their potential, given the still-prevalent negative views of the aging process. Consequently, spaces dedicated to offering structured sport practice for older adults remain limited. The findings of this study suggest that policymakers should consider public policies that facilitate older adults’ access to organized sport programs, ensuring adequate infrastructure and qualified professionals (i.e., sport coaches and managers). Furthermore, sport managers should carefully examine the characteristics of sport programs to provide genuine opportunities for older adults to engage in sport autonomously and to reap the benefits of sustained participation. To this end, they may invest in professional development opportunities for coaches, informed by the findings of this and other studies demonstrating the potential for positive development in later life, as well as implement mechanisms that enable the active listening and meaningful participation of older athletes in program planning and decision-making.

## 5. Limitations

One methodological characteristic of this study lies in the inclusion of different agents (athletes and coaches) and the use of multiple data collection strategies, such as semi-structured interviews of varying lengths and focus groups conducted across diverse contexts. While this approach may have enriched the dataset, it also introduces potential variability in the depth and nature of the information obtained. Consequently, readers should exercise caution when interpreting the findings, particularly in comparison with other studies, as these variations may have influenced the researchers’ interpretations. In addition, all participants in this study reside in the Southeast region of the country, which prevents generalization of findings to the entire population.

The volunteers were already engaged in sports for long periods, which require both time and financial resources, and, therefore, these individuals have the means to sustain their practice. We recognize that recruiting participants from vulnerable backgrounds would have enriched the study and informed the development of more targeted interventions, as certain social groups face significant barriers to accessing sport in Brazil [45,51]. Although we included people with diverse socioeconomic backgrounds and observed differences in the quality of the sport experience provided, varying downwards in more disadvantaged areas, this theme was not fully explored in this study, and, therefore, the barriers that hinder access to high-quality sport experiences promoting positive development were not fully investigated. We also acknowledge that we used a binary understanding of gender, which prevents the understanding of positive development in sport for the diversity of gender. Furthermore, we did not include people with disabilities, which would broaden the understanding of the phenomenon.

## 6. Conclusions

This study contributes to a deeper understanding of how older adults experience sport as a meaningful and largely autonomous activity that supports personal development. Participants reported high levels of engagement and satisfaction with their sport practice, primarily driven by enjoyment and personal fulfillment, suggesting a predominance of intrinsic motivation. At the same time, extrinsic motives were also present, particularly health-related goals and the influence of social encouragement to maintain an active lifestyle. Importantly, their narratives indicate that long-term sport engagement is sustained when individuals perceive themselves as competent, socially supported, and free to make choices regarding how and why they participate.

Autonomy is a central theme in these participants’ experiences. Their decisions to prioritize sport in their routines were made in balance with other life domains, reflecting an autonomous integration of sport into their identities, which is interpreted as harmonious passion, a positive outcome of these individuals’ sports practice. This engagement was fueled by a deep sense of competence and confidence developed over time and intentionally stimulated by coaches, as well as by self-awareness regarding their own skills, needs, limits, and goals. Although we interviewed individuals who already demonstrate autonomy in their sport engagement, the findings of this study suggest the importance of fostering autonomy in engagement and self-regulation of one’s own sport practice, as well as supporting the satisfaction of BPN throughout the entire sport development pathway. This may enable individuals to benefit from the positive outcomes of sport participation in later life, such as well-being, physical and mental health.

Intentional pedagogical strategies that support the satisfaction of the need for autonomy can help create sport environments that promote the development of harmonious passion for sport, are developmentally rich, and personally meaningful. Coaches are encouraged to provide structure that supports sport competence development, cultivate respectful and supportive relationships, and create opportunities for reflection on the meaning of sport participation in later life, respecting these individuals’ complexities by offering a quality sport experience based on its condition of cultural phenomenon. Emphasizing socio-educational and historical-cultural content may further contribute to reducing ageist beliefs and promoting sport as a legitimate space for growth in older adulthood, contradicting ageist assumptions that sport for this population is a space exclusively for fun or prevention of health and functional declines. When designed to respect the diverse experiences and needs of older adults, sport experience has the potential to promote positive development.

## Figures and Tables

**Table 1:** Sociodemographic characteristics of the sports practitioners participating in the study (total and per data collection group).

Data Collection Format	N	Age (M ± SD)	Sex	Race and Ethnicity	Socioeconomic Status	Educational Level	Sport Experience
Short individual or small groups interviews in a competition event	40	71.93 (±8.99)	27 W/13 M	1 As, 5 dBl, 7 lBl, 27 Wh	< 1 MW (n = 7) > 1 < 2 MW (n = 4) > 2 < 3 MW (n = 6) > 3 < 5 MW (n = 9) > 5 < 10 MW (n = 6) > 10 < 20 MW (n = 4) > 20 (n = 0) Did not answer (n = 4)	No formal education (n = 2) Elementary school (n = 8) High school (n = 11) Higher education (n = 12) Post-graduation (n = 7)	< 5 y (n = 3) 6 to 10 y (n = 7) 11 to 20 (n = 8) 21 to 30 y (n = 2) 31 to 40 y (n = 3) 41 to 60 y (n = 10) More than 60 y (n = 7)
Focus group 1	8	69.63 (±4.10)	2 W/6 M	1 As, 1 lBl, 6 Wh	7 ≤ 1 MW (n = 0) > 1 < 2 MW (n = 1) > 2 < 3 MW (n = 2) > 3 < 5 MW (n = 3) > 5 < 10 MW (n = 1) > 10 < 20 MW (n = 0) > 20 (n = 0) Did not answer (n = 1)	No formal education (n = 0) Elementary school (n = 2) High school (n = 2) Higher education (n = 2) Post-graduation (n = 0) Did not answer (n = 2)	< 5 y (n = 2) 6 to 10 y (n = 1) 11 to 20 (n = 1) 21 to 30 y (n = 0) 31 to 40 y (n = 0) 41 to 60 y (n = 0) More than 60 y (n = 4)
Focus group 2	9	76.89 (±6.25)	3 W/6 M	9 Wh	7 ≤ 1 MW (n = 0) > 1 < 2 MW (n = 0) > 2 < 3 MW (n = 1) > 3 < 5 MW (n = 1) > 5 < 10 MW (n = 0) > 10 < 20 MW (n = 4) > 20 (n = 3) Did not answer (n = 0)	No formal education (n = 0) Elementary school (n = 0) High school (n = 1) Higher education (n = 5) Post-graduation (n = 3) Did not answer (n = 0)	< 5 y (n = 0) 6 to 10 y (n = 0) 11 to 20 (n = 1) 21 to 30 y (n = 0) 31 to 40 y (n = 0) 41 to 60 y (n = 1) More than 60 y (n = 7)
Focus group 3	7	77.14 (±4.22)	7 W	7 Wh	7 ≤ 1 MW (n = 0) > 1 < 2 MW (n = 1) > 2 < 3 MW (n = 2) > 3 < 5 MW (n = 3) > 5 < 10 MW (n = 0) > 10 < 20 MW (n = 1) > 20 (n = 0) Did not answer (n = 0)	No formal education (n = 0) Elementary school (n = 1) High school (n = 3) Higher education (n = 1) Post-graduation (n = 1) Did not answer (n = 1)	< 5 y (n = 0) 6 to 10 y (n = 1) 11 to 20 y (n = 0) 21 to 30 y (n = 1) 31 to 40 y (n = 0) 41 to 60 y (n = 4) More than 60 y (n = 1)
Focus group 4	8	69.25 (±7.38)	8 M	1 As, 1 lBl, 6 Wh	7 ≤ 1 MW (n = 0) > 1 < 2 MW (n = 0) > 2 < 3 MW (n = 0) > 3 < 5 MW (n = 4) > 5 < 10 MW (n = 2) > 10 < 20 MW (n = 2) > 20 (n = 0) Did not answer (n = 0)	No formal education (n = 0) Elementary school (n = 0) High school (n = 1) Higher education (n = 6) Post-graduation (n = 0) Did not answer (n = 1)	< 5 y (n = 2) 6 to 10 y (n = 0) 11 to 20 (n = 3) 21 to 30 y (n = 0) 31 to 40 y (n = 0) 41 to 60 y (n = 3) More than 60 y (n = 0)
Focus group 5	8	66.63 (±4.41)	6 W	2 As, 1 dBl, 5 Wh	< 1 MW (n = 0) > 1 < 2 MW (n = 1) > 2 < 3 MW (n = 1) > 3 < 5 MW (n = 2) > 5 < 10 MW (n = 0) > 10 < 20 MW (n = 3) > 20 (n = 1) Did not answer (n = 0)	No formal education (n = 0) Elementary school (n = 0) High school (n = 1) Higher education (n = 5) Post-graduation (n = 2) Did not answer (n = 0)	< 5 y (n = 0) 6 to 10 y (n = 1) 11 to 20 (n = 6) 21 to 30 y (n = 0) 31 to 40 y (n = 0) 41 to 60 y (n = 0) More than 60 y (n = 1)
Total	80	71.91 (±7.91)	45 W	5 As, 6 dBl, 9 lBl, 60 Wh	< 1 MW (n = 7) > 1 < 2 MW (n = 7) > 2 < 3 MW (n = 12) > 3 < 5 MW (n = 22) > 5 < 10 MW (n = 9) > 10 < 20 MW (n = 14) > 20 (n = 4) Did not answer (n = 5)	No formal education (n = 2) Elementary school (n = 11) High school (n = 19) Higher education (n = 31) Post-graduation (n = 13) Did not answer (n = 4)	< 5 y = 7 6 to 10 y = 9 11 to 20 y = 20 21 to 30 y = 3 31 to 40 y = 3 41 to 60 y = 18 More than 60 y = 20

Notes: As—Asian, dBl—Dark Black, lBl—Light-Black, Wh—White, MW—minimum wage, y—years, W—women, n—number of participants.

**Table 2:** Sociodemographic characteristics of the coaches participating in the study.

Name (Fictitious)	Age	Education	Race/Ethnicity	Experience with Master Athletes (Years)	Main Sport Coached
Laura	52	Bachelor’s and master’s degrees in PE	lBl	24	Swimming
Rafael	57	Bachelor’s and master’s degrees in PE	Wh	41	Adapted volleyball
Tadeu	50	Bachelor’s degree and lato sensu specialization in PE	Not reported	16	Adapted volleyball
Fernando	37	Bachelor’s degree in PE	Wh	13	Badminton

Note: PE = physical education, lBl—Light-Black, Wh—White.

## Data Availability

The data presented in this study are available on reasonable request from the corresponding author due to ethical reasons related to the preservation of participants’ identities.

## Abbreviations

The following abbreviations are used in this manuscript:

PDS	Positive Development in Sport
SDT	Self-Determination Theory
BPN	Basic Psychological Need
Wh	White
lBl	Light-Black
dBl	Dark-Black
As	Asian
MW	Minimum Wage
Y	Year
PE	Physical Education
JOMI	Municipal Older People Game
DMSP	Developmental Model of Sport Participation
WHO	World Health Organization
